# Metabolomics approach to assess the effect of siphonal autotomy on metabolic characteristics of razor clam *Solen grandis*

**DOI:** 10.1038/s41598-022-09562-6

**Published:** 2022-04-01

**Authors:** Yangping Wu, Aihua Chen, Yu Zhang, Zhidong Zhang, Yi Cao, Suhua Chen, Zhen Tian, Qiujie Li

**Affiliations:** 1 Marine Fisheries Institute of Jiangsu Province, Nantong, 226007 China; 2 Jiangsu Fine Breeding Farm of Solen Grandis, Nantong, 226007 China

**Keywords:** Animal physiology, Ichthyology

## Abstract

Autotomy appendages are fundamental evolutionary adaptations to escape predation. The siphon is an important foraging organ for bivalves. Here, we report the first demonstration of autotomy of the siphon in marine bivalves (razor clam *Solen grandis*) and the effect of siphonal autotomy in *S. grandis* on foraging and metabolic characteristics. In this study, the feeding rate and digestive enzyme activities upon siphonal autotomy in razor clams were investigated. Moreover, endogenous metabolites pre/post-autotomy of the siphon were investigated using liquid chromatography tandem-mass spectrometry (LC–MS). The feeding rate and digestive enzyme activities decreased significantly after siphonal autotomy in *S. grandis* (*P* < 0.05), suggesting that autotomy of the siphon negatively affected its foraging. These results might be related to the reduction in the foraging radius. Additionally, the effect of autotomy was investigated on a total of 34 differentially abundant metabolites, and pathway analysis indicated that 32 differentially enriched metabolic pathways were worthy of attention. Further integrated key metabolic pathway analysis showed that glycine, serine and threonine metabolism; taurine and hypotaurine metabolism; biotin metabolism; vitamin B6 and thiamine metabolism were significantly relevant pathways in *S. grandis* pre/post-autotomy of the siphon. The downregulation of glycine, taurine, and hypotaurine is expected to indicate a shortage of intermediate compounds and energy in *S. grandis*. Therefore, to provide the required energy and materials for siphon regeneration in *S. grandis*, we anticipated that it would be necessary to supplement these as exogenous metabolites from the daily diet.

## Introduction

Autotomy (self-amputation), an animal’s ability to shed a body part without any external force, is a common anti-predator behavior^1,2^. Autotomy occurs in a wide range of taxa, such as echinoderms, reptiles, arthropods, and crustaceans^3–6^. To date, it has been most frequently studied in lizards where their tails are shed in response to a predator^7,8^. After autotomy, the loss of appendages is permanently lost in mammals. However, autotomized appendages may be replaced by regeneration in many (but not all) animals^9^. Although autotomy appendages provide direct fitness benefits to avoid being killed or poisoned, autotomy can reduce function, which may negatively affect competition, mobility, mating and foraging^10,11^. Previous studies have shown that autotomizing geckos are significantly slower than intact geckos during escape^12,13^. In line with this, after autotomy of lamellae, larvae of the damselfly *Lestes viridis* showed low levels of innate immunity (phenol oxidase) and antioxidant defense (superoxide dismutase)^14^. Similarly, autotomy of the claw could stimulate molting and suppress feeding in fiddler crabs^15^. Here, we report the first demonstration of autotomy of the siphon in marine bivalves (razor clam *Solen grandis*) and the effect of siphonal autotomy in *S. grandis* on foraging and metabolic characteristics.

*Solen grandis*, commonly known as the razor clam, is a long bivalve that is naturally distributed along the west coast of the Pacific Ocean^16,17^. In China, *S. grandis* is regarded as a precious seafood because of its delicious taste and high nutritional content^18^. However, the number of *S. grandis* has been severely declining owing to changes in the natural environment and overfishing in the last decade^19,20^. Recently, *S. grandis* has been farmed by pond culture. In the process of aquaculture, razor clam siphons are prone to autotomy because of external stimuli, such as tides and the agitation of symbiotic organisms. Furthermore, after autotomy, the clam is weak and may even die and cannot dive deep into the sand, which has a serious effect on the survival rates of razor clams. In addition, collision and shaking may cause razor clams to autotomize siphons under stimulated conditions during transportation and sales, causing the razor clams to lose body weight and price. However, the effects of siphonal autotomy and the underlying metabolic characteristics of *S. grandis* remain poorly understood.

Metabolomics is an emerging technological and analytical approach for comprehensively analyzing the various metabolites contained in biological samples^21,22^. Metabolites comprise all compounds in a biological matrix that are typically smaller than 1 kDa in size and include small peptides, oligonucleotides, sugars, organic acids, ketones, aldehydes, amino acids, lipids, steroids, alkaloids and xenobiotics^23,24^. With the development of metabolomics technology, many tools are currently available, including liquid chromatography tandem-mass spectrometry (LC–MS) and gas chromatography–mass spectrometry (GC–MS)^23^. However, compared with GC–MS, LC–MS has a high peak capacity, resolution, and sensitivity. It is suitable for the analysis of metabolites with a high boiling point, high molecular weight or limited thermal stability^23,25^. Thus, LC–MS is highly suitable for the detection of a wide array of metabolites. To date, LC–MS metabolomics have been applied to metabolic studies of bivalves. For instance, Tian et al. reported that the metabolic characteristics of live scallops (*Mizuhopecten yessoensis*) subjected to mechanical shock were investigated in the early post-harvest process^22^. Sun et al. revealed the molecular responses of clams to acute hypoxia by combining integrated transcriptome and metabolome (LC–MS) analysis^26^. Abraham et al. identified biomarkers of brevetoxin exposure in hard clams (*Mercenaria sp.*) exposed to *Karenia brevis* blooms using LC-MS^27^. However, to the best of our knowledge, there are no studies that have investigated the metabolic characteristics of *S. grandis*.

In this study, we investigated the feeding rate and digestive enzyme activities upon siphonal autotomy in razor clams. Moreover, using LC–MS, we explored the changes in endogenous metabolites pre/post-autotomy of the siphon. The results contribute to data on the underlying metabolic characteristics and toward improving aquaculture of *S. grandis*.

## Materials and methodology

### Sample collection and preparation

Razor clams were obtained from the Jiangsu Fine Breeding Farm of *Solen grandis* (Nantong, China). A total of 18 individuals were randomly selected, including 9 razor clams with induced autotomy of the siphon and 9 razor clams with a complete siphon. Three individuals were placed in one aquarium were considered a single sample. Therefore, a total of six samples were divided into two groups: three pre-autotomy groups and three post-autotomy groups. The feeding rates of *S. grandis* were assessed by measuring the volume of water cleared from suspended microalgae (*Isochrysis galbana*) per hour^28^. At the end of the feeding experiment, we measured body weight and dissected the digestive glands and siphon base separately for each sample. Each sample was washed with 0.01 M phosphate buffered saline, immediately frozen in liquid nitrogen, and stored at − 80 ℃ for subsequent metabolite isolation and bioactivity determination.

### Determination of feeding rates and digestive enzyme activity assay

The feeding rate was measured by means of the clearance method using an aquarium with three *S. grandis* and well-mixed seawater with added algal cells (*Isochrysis galbana*) that are 100% efficiently retained by the gills of *S. grandis*^28^. The algal concentration in seawater was measured using a hemocytometer both at the beginning and end (1 h later) of the experiment. The feeding rate was determined from the exponential decrease in algal concentration as a function of time.

The digestive glands were weighed and homogenized in ice-cold 0.86% sterile saline solution (tissue: saline, 1:9). Then, the homogenates were centrifuged at 1200 g at 4 ℃ for 15 min, and the supernatants were collected and stored at − 80 ℃ for the analysis of digestive enzymes. The activities of digestive enzymes, including protease, amylase, and lipase, were examined spectrophotometrically using commercial assay kits from Nanjing Jiancheng Bioengineering Institute (Nanjing, China) according to the manufacturer’s instructions^29^. All assays were performed in triplicate. In the reaction, protease hydrolyzes proteins to produce phenolic amino acids, and phenolic reagents can be reduced to blue substances by phenolic amino acids. Thus, one unit of protease was defined as the production of 1 μg of amino acid per milligram of histone per minute at 37 °C. Amylase can hydrolyze starch to generate glucose, maltose, and dextrin. In the case of known and excessive substrate concentrations, iodine solution is added to combine with unhydrolyzed starch to generate a blue complex. Amylase activity was calculated according to the depth of blue color. One amylase activity unit was defined as the hydrolysis of 10 mg of starch per mg protein at 37 °C for 30 min. Latex made of triglycerides and water has opacification properties owing to the absorption and scattering of incident light by micelles. Under the action of lipase, the triglycerides in micelles hydrolyze, causing micelles to split, thus reducing the scattering light or turbidity. The rate of decrease is related to lipase activity. One unit of lipase was defined as each gram of tissue protein reacting with each substrate consumed 1 μmol substrate per minute in the reaction system at 37 °C. It is worth noting that the activities of digestive enzymes in this study are presented as specific activities.

### Metabolite extraction and analysis for LC–MS

We dissected the siphon base tissue to measure the metabolites of the razor clam *S. grandis*. Twenty-five milligrams of sample were weighed and placed into a clean microcentrifuge tube, and 500 µL of extraction solution (acetonitrile: methanol: water = 2: 2: 1) containing isotopically labeled internal standard mixture was added. After vortexing for 30 s, the samples were homogenized at 35 Hz for 4 min (JXFSTPRP-24; Shanghai Jingxin Technology Co., Ltd., China) and sonicated for 5 min in an ice-water bath (YM-080S, Shenzhen Fangao Microelectronics Co., Ltd., China). Homogenization and sonication cycles were repeated for twice. The samples were incubated at − 40 ℃ for 1 h and centrifuged at 13,800 g for 15 min at 4 ℃ (Heraeus Fresco17 series; Thermo Fisher Scientific Inc., USA). The supernatant (400 μL) was transferred to a fresh tube and dried in a vacuum concentrator at 37 ℃. Then, the dried samples were reconstituted in 200 μL of 50% acetonitrile by sonication for 10 min in ice-water bath. The mixture was then centrifuged at 13,800 g for 15 min at 4 ℃, and 75 μL of supernatant was transferred to a fresh glass vial for LC–MS analysis. The quality control sample was prepared by mixing an equal aliquot of the supernatant from all samples.

The UHPLC separation was performed using a 1290 Infinity series UHPLC System (Agilent Technologies, USA), equipped with a UPLC BEH Amide column (2.1 × 100 mm, 1.7 μm, Waters)^24^. The mobile phases consisted of 25 mmol/L ammonium acetate and 25 mmol/L ammonia hydroxide in water (pH = 9.75) (A) and acetonitrile (B). The analysis was carried out with an elution gradient as follows: 0–0.5 min, 95%B; 0.5–7.0 min, 95–65% B; 7.0–8.0 min, 65–40% B; 8.0–9.0 min, 40% B; 9.0–9.1 min, 40–95% B; 9.1–12.0 min, 95% B. The column temperature was maintained at 25 ℃. The auto-sampler temperature was 4 ℃, and the injection volumes were 2 µL for both positive (pos) and negative (neg) samples.

Triple TOF 6600 mass spectrometry (AB SCIEX) was used for its ability to acquire MS/MS spectra on an information-dependent basis during an LC–MS experiment. In this mode, the acquisition software (Analyst TF 1.7; AB Sciex) continuously evaluates the full scan survey MS data as it collects and triggers the acquisition of MS/MS spectra depending on preselected criteria. In each cycle, the most intensive 12 precursor ions with intensity above 100 were chosen for MS/MS at a collision energy of 30 eV. The cycle time was 0.56 s. The ESI source conditions were set as following: Gas 1 as 60 psi, Gas 2 as 60 psi, curtain gas as 35 psi, source temperature as 600 ℃, declustering potential as 60 V, ion spray voltage floating as 5000 V or − 4000 V in positive or negative modes, respectively.

### Statistical analysis

The MS raw data (.wiff) files were converted to the mzXML format using ProteoWizard, and processed using R package XCMS^30^. The process includes peak deconvolution, alignment and integration. Minfrac and cut off were set as 0.5 and 0.3 respectively. The in-house MS2 database was used for metabolite identification. The significance of the differences between groups was determined using Student’ s *t*-test. Body weight, feeding rate, and digestive enzyme activities are presented as the mean ± SD.

## Results

### Comparison of feeding rate and digestibility

**Figure 1:**
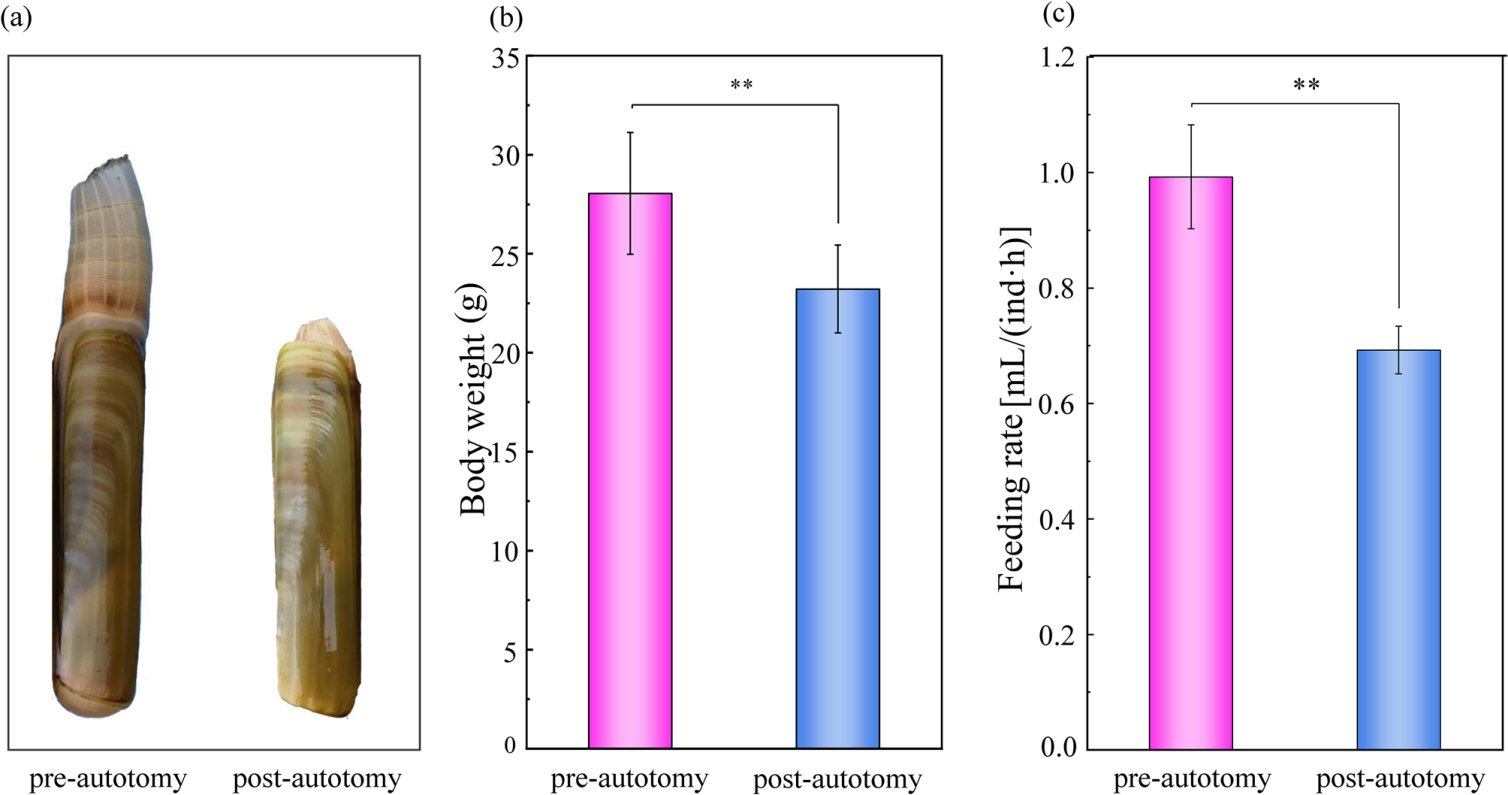
Effect of siphonal autotomy on body weight and feeding rate in *Solen grandis*. Data are expressed as mean ± SD. The asterisk (**) indicates significant difference at *P* < 0.01.

**Figure 2:**
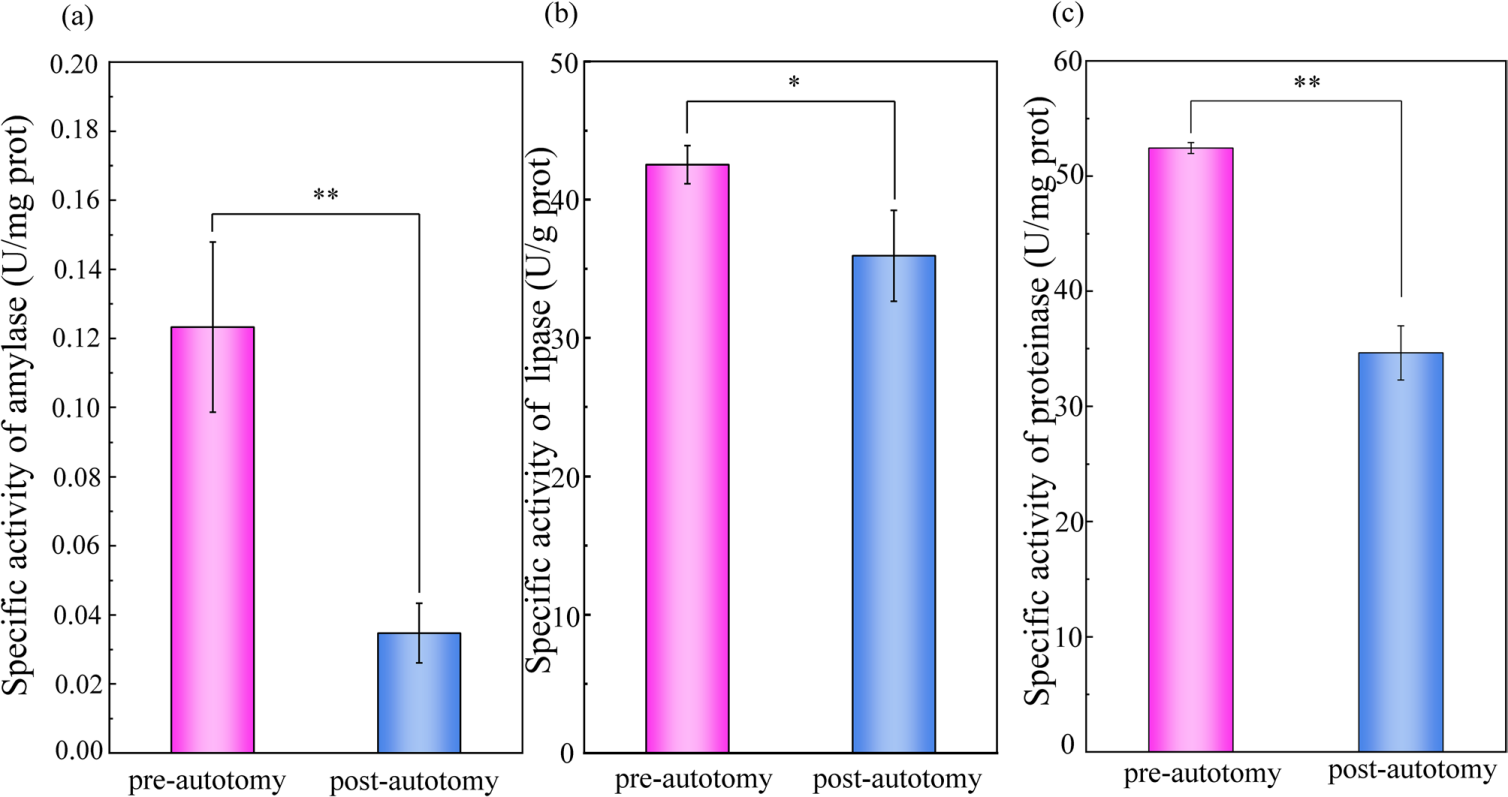
Effect of siphonal autotomy on digestive enzyme activities in *Solen grandis*. Data are expressed as mean ± SD. The asterisk (*) and (**) indicate a significant difference at *P* < 0.05 and *P* < 0.01, respectively.

The body weight was reduced by 4.83 ± 0.85 g and the feeding rate of *S. grandis* was down-regulated significantly (*P* = 0.035) after siphonal autotomy (Fig. 1). To further understand the effect of siphonal autotomy on feeding capacity, digestive enzyme activity levels were measured. The study of amylase, lipase, and proteinase activities pre/post-autotomy of the siphon can help us understand the food digestion capacity of *S. grandis*. In this study, digestive enzyme activity levels in the post-autotomy group were significantly lower than in the pre-autotomy group (*P* < 0.05), suggesting that the digestibility in *S. grandis* was significantly inhibited after autotomy (Fig. 2).

### Metabolic profiles analyzed by LC–MS

**Figure 3:**
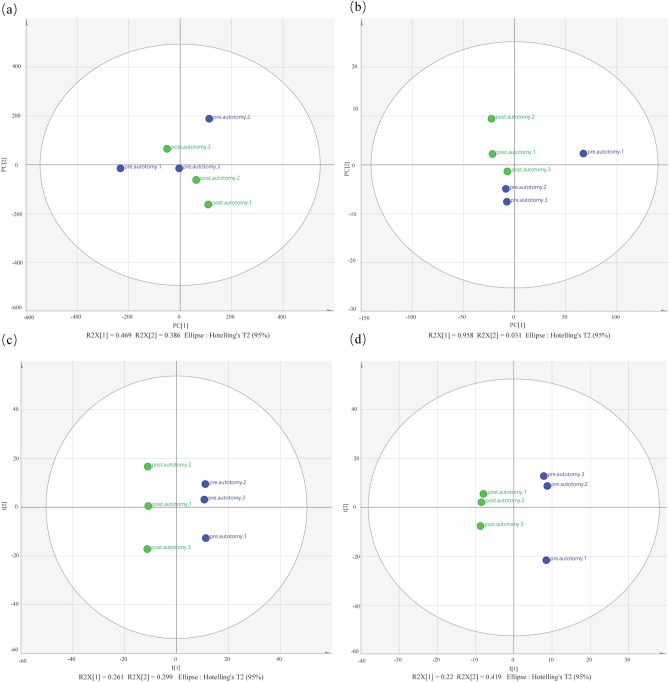
Principal component analysis (PCA) and orthogonal projections to latent structures–discriminant analysis (OPLS-DA) score plots in liquid chromatography tandem-mass spectrometry (LC–MS) metabolite profiles pre/post autotomy of the siphon in *Solen grandis*. (**a**,**b**) PCA score plots and (**c**,**d**) OPLS-DA score plots. Left (**a**,**c**): positive ion mode and right (**b**,**d**): negative ion mode.

All metabolites were analyzed using unsupervised principal component analysis (PCA). PCA score results showed that the pre/post-autotomy data points were significantly separated in spatial distribution and the R^2^X values of the PCA model accounting for the variance were 0.469 and 0.958 in the positive and negative ion modes, respectively (Fig. 3a,b). To maximize the discrimination between the two groups, we employed orthogonal projections to latent structures–discriminant analysis (OPLS–DA) to elucidate the different metabolic patterns. The OPLS–DA results show that data points between the two groups were divided into separate clusters, suggesting that metabolic patterns pre/post-autotomy of the siphon in *S. grandis* were significantly different (Fig. 3c,d). All samples in the score plots were within the 95% Hotelling’s T-squared ellipse in PCA and OPLS-DA, thereby indicating that there were no outliers among the analyzed samples and might be exploited in subsequent analyses.

### Changed metabolites pre/post-autotomy of the siphon in *S. grandis*

**Figure 4:**
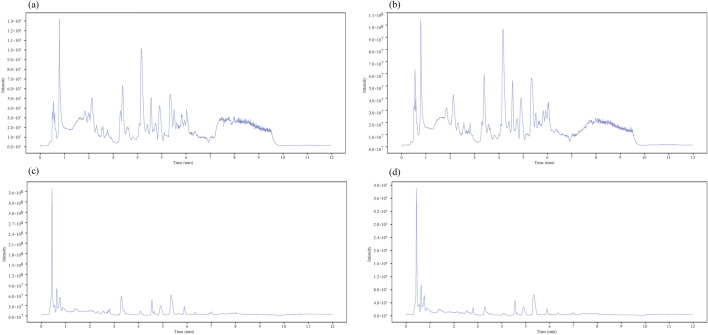
Typical total ion chromatography of siphon extracts obtained from (**a**,**b**) positive ion (POS) and (**c**,**d**) negative ion (NEG) modes in liquid chromatography tandem-mass spectrometry (LC–MS). Left (**a**,**c**): pre-autotomy samples and right (**b**,**d**): post-autotomy samples.

**Figure 5:**
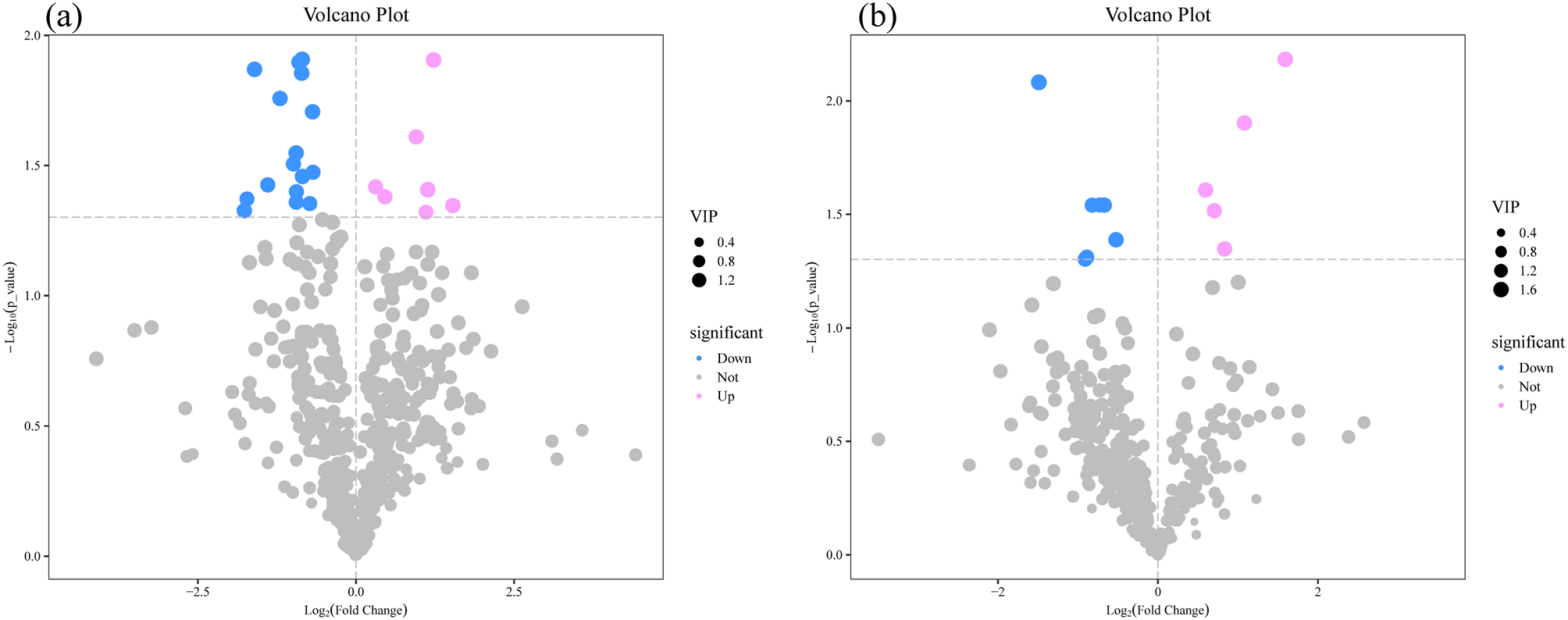
Volcano plots of differential metabolites were derived from (**a**) positive ion (POS) and (**b**) negative ion (NEG) modes. Each point represents a metabolite, and the point size represents the VIP value of this metabolite in the orthogonal projections to latent structures–discriminant analysis (OPLS-DA) model. Red dots indicate significantly upregulated metabolites (VIP > 1 and *P* < 0.05). Blue dots indicate significantly downregulated metabolites (VIP > 1 and *P* < 0.05). Gray dots indicate no significant difference pre/post autotomy of the siphon in *Solen grandis* (VIP ≤ 1 or *P* ≥ 0.05).

**Figure 6:**
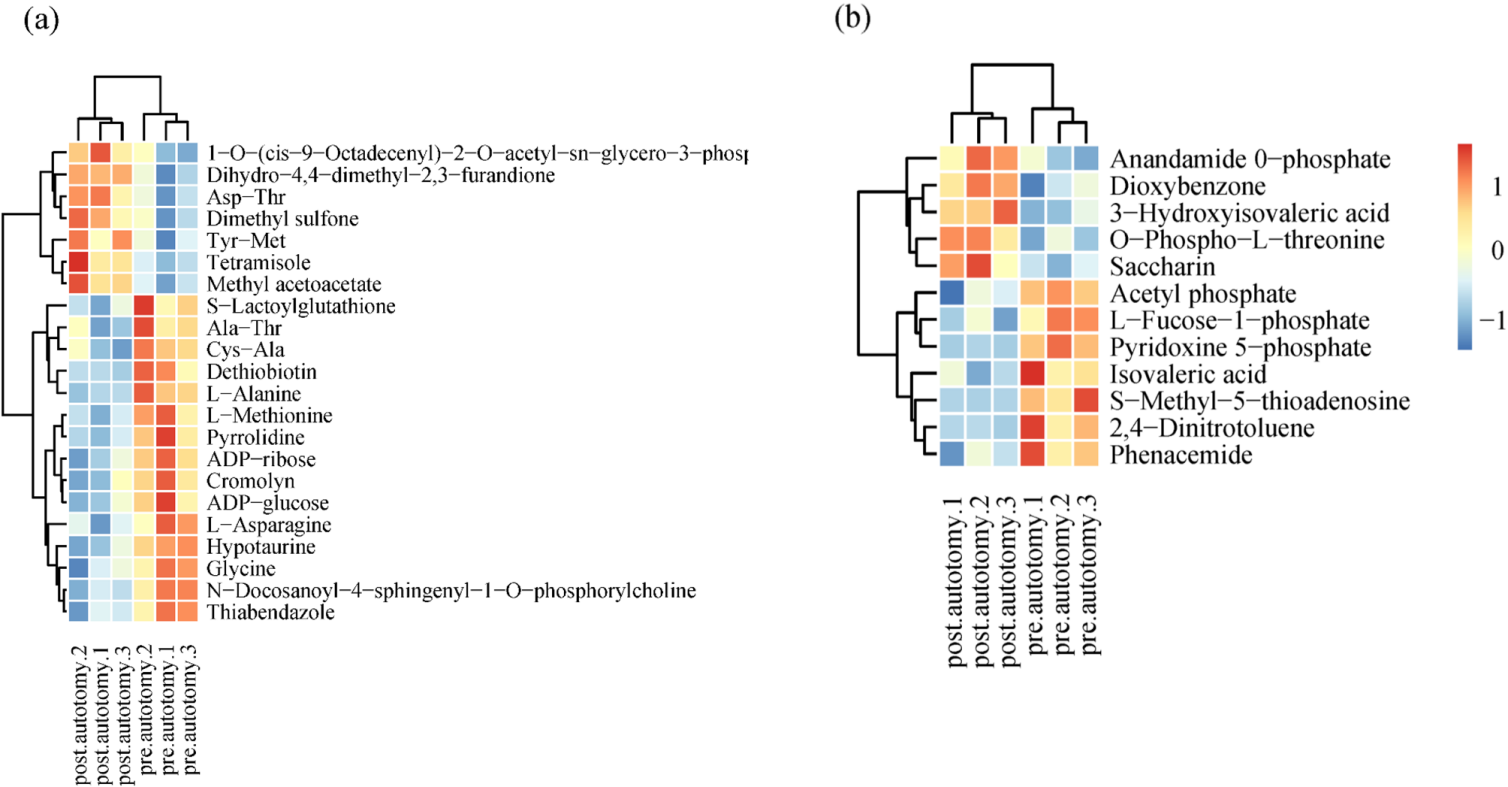
Hierarchical clustering analysis thermal map based on significantly differential metabolites (SDMs) from (**a**) positive ion (POS) and (**b**) negative ion (NEG) modes. The relative metabolite level is depicted according to the color scale; red and blue indicate upregulation and downregulation, respectively.

The typical LC–MS total ion chromatography of samples from the pre/post-autotomy groups are shown in Fig. 4. A total of 7135 (POS:3731, NEG:3404) peaks were deconvoluted using LC–MS. The shape and number of peaks were different, reflecting the difference in metabolite spectra pre/post-autotomy of the siphon in *S. grandis*. In contrast, only 938 (POS:559, NEG:379) remaining metabolite peaks were further annotated using references in existing databases after filtering and denoising of LC–MS data. Fold Change values were used to represent specific variables pre/post autotomy of the siphon in *S. grandis*. The distribution of metabolites was divided into upregulated and downregulated metabolites according to the fold Change values. Volcano plots were used to illustrate the relationships between the *P*-value and fold change of all the identified metabolites, representing the degree of difference and the statistical significance in the pre/post autotomy groups in *S. grandis*. The volcano plots showed that 34 significantly differential metabolites (SDMs) (VIP > 1 and *P* < 0.05) were determined pre/post autotomy of the siphons in *S. grandis*, based on the OPLS–DA results. Among these SDMs, 22 differential metabolites were identified based on the positive ion mode, including seven upregulated metabolites and 15 downregulated metabolites (Fig. 5a). Based on the negative ion mode, 12 differential metabolites were identified, including five upregulated metabolites and seven downregulated metabolites (Fig. 5b). These SDMs include carbohydrate metabolites (e.g., ADP-ribose, and ADP-glucose), amino acids and their derivatives (e.g., glycine, asparagine, and alanine.), lipid metabolites (e.g., dethiobiotin, methyl acetoacetate, and hypotaurine), and other metabolites (e.g., tetramisole, and thiabendazole) (Fig. 6).

### Characterization and functional analysis of key metabolic pathways of significantly differential metabolites

**Figure 7:**
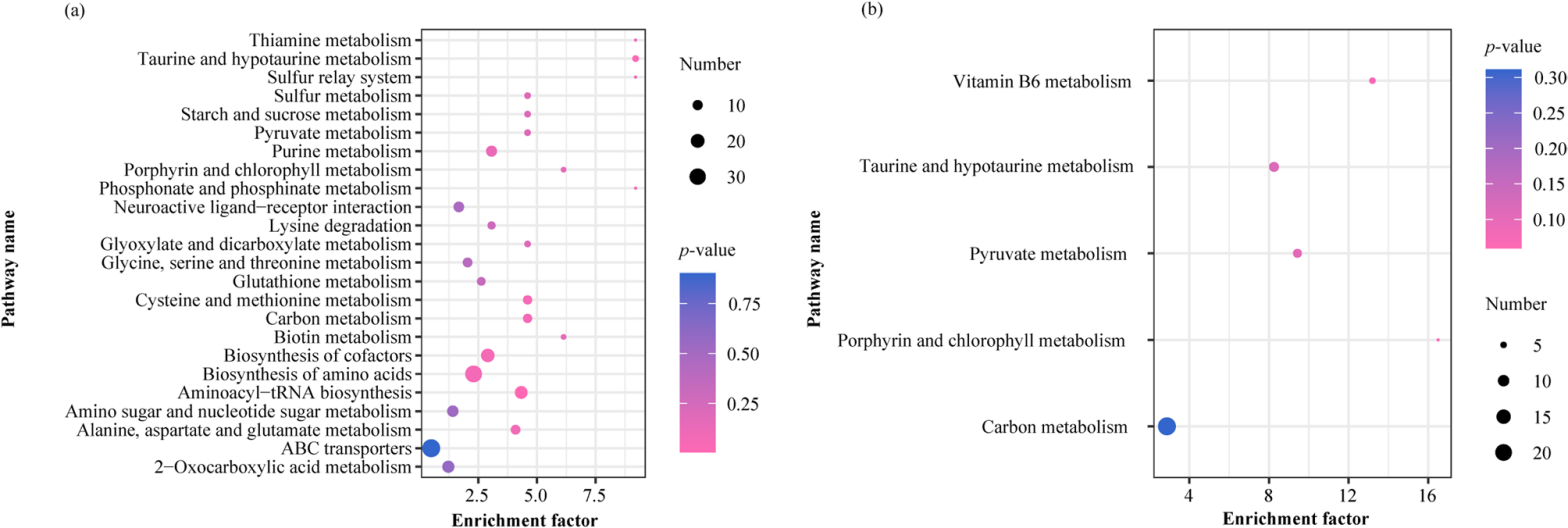
Statistical scatter plot of pathway enrichment of differential metabolites. (**a**) Positive ion (POS) and (**b**) negative ion (NEG) modes; the *x*-axis and *y*-axis represent enriched metabolic pathways and enrichment factors, respectively; rich factor refers to the ratio of differentially expressed metabolites to all metabolites annotated to this metabolic pathway; the larger the rich factor, the greater is the degree of enrichment. *P*-value, ranging from 0 to 0.05; the closer to 0, the more significant is the enrichment.

**Figure 8:**
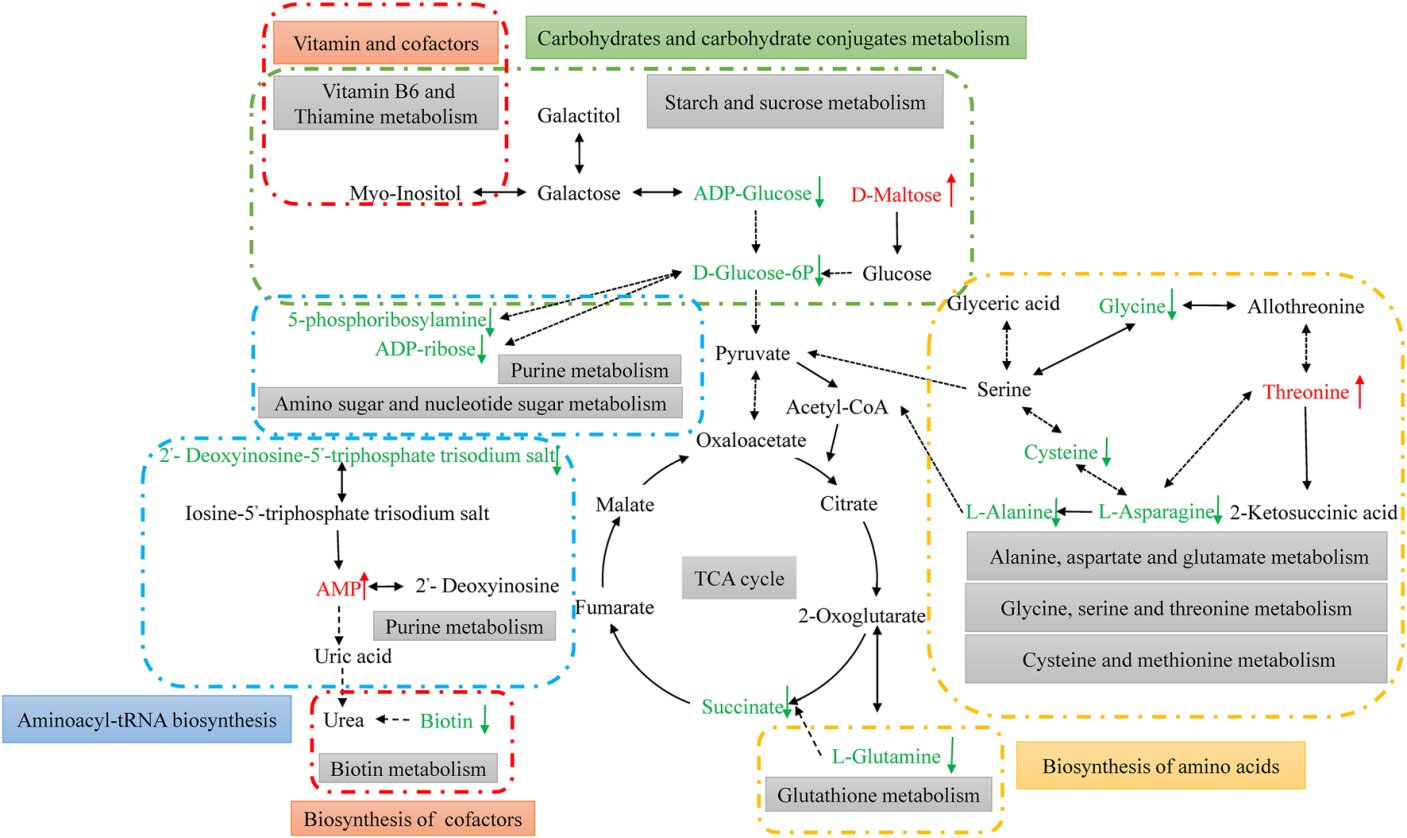
Schematic overview of the primarily metabolic pathways affected autotomy of siphon among *S. grandis*. The red characters indicated increased metabolites, and the green ones indicated decreased metabolites.

SDMs were imported into MetaboAnalyst 4.0 and KEGG pathway analysis was performed to identify the potential metabolic pathways that are affected upon autotomy of the siphon in *S. grandis*^31^. A total of 32 pathways (POS:26, NEG:6) were identified (Fig. 7). We constructed a schematic overview based on the reference diagrams stored in the KEGG database^32,33^ (Fig. 8). Base on both the enrichment factor and pathway name, the relevant metabolic pathways were identified as vitamin B6 and thiamine metabolism; alanine, aspartate and glutamate metabolism; starch and sucrose metabolism; and taurine and hypotaurine metabolism (Fig. 7). We summarized these different enriched metabolic pathways as the metabolism of amino acids, carbohydrates, nucleosides, vitamins and cofactors.

## Discussion

Autotomy (self-amputation) may negatively affect competition, mobility, mating and foraging^3–6^. The siphon is an important foraging organ for bivalves. In this study, the feeding rate and digestion capacity decreased after siphonal autotomy in *S. grandis*, which may be related to the reduction in foraging radius. However, the material changes and accumulation in these bivalves remain poorly understood. Importantly, determination of the metabolite changes inside the cells after autotomy of the siphon will help us provide new insights into the underlying metabolic characteristics and help improve aquaculture of *S. grandis*.

In total, 32 major pathways were observed pre/post-autotomy of the siphon in *S. grandis*, of which amino acid metabolism was significant and played a significant role in autotomy. Glycine is regarded as a key link to one‑carbon metabolism and is involved in the methylation of proteins and deoxyribonucleic acid^34^. It is also a fundamental component of the biosynthesis of heme, purines, creatine, glutathione, and uric acid^35^. The relatively low content of glycine after autotomy of the siphon could be related to L-alanine, L-aspartate, and L-methionine metabolism. These nitrogenous metabolites are further converted into intermediates of the TCA cycle to compensate for the shortage of energy and intermediate compounds (e.g., Cys-Ala, and Ala-Thr). Taurine and hypotaurine were the two SDMs in the TCA cycle in this study. Taurine is known to maintain cell membrane permeability in calcium homeostasis^36^. Taurine can also remove oxidizing free radicals and plays a strong antioxidant role in detoxification and osmoregulation^37^. When the razor clam’s siphon undergoes autotomy, a large amount of oxidized free radicals may be produced. However, the razor clam has the ability to respond to autotomy by changing taurine and hypotaurine metabolism. The TCA cycle has two important functions. The first is an intermediate compound that synthesizes amino acids and fatty acids. The second involves the formation of large amounts of ATP, which provide energy for various biosynthetic processes^38^. The downregulation of glycine, taurine and hypotaurine in *S. grandis* is expected to indicate that a shortage of intermediate compounds and energy^37^, which may be relevant to the hunger induced by the autotomy. Many studies have shown that a shortage of endogenous metabolites can be compensated for by external diet^39,40^. Therefore, it is necessary to supplement with exogenous metabolites in the daily diet to maintain the regeneration of the siphon in *S. grandis*.

It is well established that several amino acids are converted into intermediates in the TCA cycle. Following activation of the anaplerotic process by autotomy, the concentrations of amino acids involved in this process change. In this study, glycine, cysteine, asparagine, and alanine were significantly decreased, whereas threonine levels were significantly increased after autotomy. Threonine is reversibly catalyzed to asparagine and allothreonine after autotomy of the siphon in *S. grandis*. Glutamine is also catalyzed to succinate via the glutathione metabolic pathway. Therefore, these pathways appear to be catalyzed to decrease the levels of the TCA cycle by autotomy.

Autotomy also affected carbohydrate metabolism in *S. grandis*. For energy production, glycolysis is initiated from glucose 6-phosphate, which is phosphorylated glucose. In the present study, glucose 6-phosphate levels significantly decreased after autotomy. Similarly, the levels of ADP-glucose, ADP-ribose, and 5′-phosphoribosyl amine were also significantly decreased after autotomy. Figure 8 shows that the concentrations of these metabolites were relatively lower than those of other metabolites involved in the glycolytic and purine and nucleotide sugar metabolic pathways. Additionally, there is a shortage of intermediate compounds and energy caused by the downregulation of succinate. The relatively low carbohydrate content observed in this study can be attributed to the glucose and glucose conjugates that break down maltose being converted into TCA cycle intermediates^41^. Therefore, such changes in sugar levels are expected to affect the generation of energy and intermediate compounds in order to maintain normal biological processes^21^. Glycose may also be a key factor in promoting the regeneration of the siphon of razor clams.

In addition, autotomy affected the vitamin and cofactor metabolism of *S. grandis*, including dethiobiotin (vitamin B7), vitamin B6 and thiamine (vitamin B1). The dethiobiotin content decreased significantly after autotomy of the siphon. This is because cofactors play critical roles in the intermediate metabolism of gluconeogenesis, amino acid catabolism and fatty acid biosynthesis^42,43^. Hence, targeted metabolomics for the vitamin B-complex family should be considered to explore the effect of autotomy on the vitamins of *S. grandis*.
